# Tick-borne encephalitis virus in *Dermacentor reticulatus* ticks, Poland

**DOI:** 10.7717/peerj.21588

**Published:** 2026-08-10

**Authors:** Drew David Reinbold-Wasson, Jaykumar Gandhi, Tao Li, Ozan L. Z. Kiratli, Joshua B. Richardson, Jeffrey R. Kugelman, Zebulon R. Lapoint, Peter J. Bernota, Grant R. Hall, Christian K. Fung, Heather L. Friberg, Gregory D. Gromowski, Michael K. McCracken, Giorgi Kirkitadze, Anano Shubashishvili, Tamar Chunashvili, Natalie D. Collins, Thomas A. Musich, Jun Hang, Matthew A. Conte

**Affiliations:** 1 Walter Reed Army Institute of Research-Europe and the Middle East, Tbilisi, Georgia; 2 Viral Diseases Program, Walter Reed Army Institute of Research, Silver Spring, Maryland, United States; 3 Center for Genome Science, US Army Medical Research Institute of Infectious Diseases, Frederick, Maryland, United States; 4 Cherokee Federal, Tulsa, Oklahoma, United States; 5 Chenega Corporation, Anchorage, Alaska, United States

**Keywords:** Tick surveillance, Viral surveillance, Tick-borne encephalitis virus (TBEV), *Dermacentor reticulatus*, Poland, Genome sequencing, Phylogenetic analysis

## Abstract

We report the detection of tick-borne encephalitis virus (TBEV) in *Dermacentor reticulatus* ticks collected in Poland during the 2022 season. Both *Ixodes ricinus* and *D. reticulatus* ticks were surveilled. There was limited TBEV detection found in one pool out of 106 pools (418 ticks) that were shotgun genome sequenced. The TBEV positive tick pool consisted of *N* = 3 adult *D. reticulatus* ticks. Additional hybrid-capture sequencing of this pool resulted in the first full-length genome sequence of TBEV from this tick species. The assembled genome was classified as the European subtype through phylogenetic analysis. The new TBEV sequence is phylogenetically placed closest to a 2019 Hungarian isolate, suggesting potential viral migration or shared ancestry across nearly 1,000 km. These findings expand the known geographic range of TBEV circulation in Poland and highlight the role of *D. reticulatus* as an important vector of TBEV.

## Introduction

Tick-borne encephalitis (TBE) is a significant human disease caused by *Orthoflavivirus encephalitidis* also commonly referred to as Tick-borne encephalitis virus (TBEV), a flavivirus transmitted by ticks. TBEV disease severity can range from asymptomatic to cases with meningitis, encephalitis, meningoencephalitis, and case fatality rates of 2–40% depending on the infecting subtype (Bogovic & Strle, 2015). TBEV infections can cause neurological damage and long-term neurological sequelae (Halsby et al., 2025). The disease is endemic across large parts of Europe and Asia, resulting in substantial morbidity and mortality (Gritsun, Lashkevich & Gould, 2003). TBE has regional specific incidence variation from <1/100,000 to 10/100,000 inhabitants (Mittova et al., 2024). TBE is endemic in Baltic countries with higher incidence: Estonia 10.1/100,000, Latvia (11.7/100,000), and Lithuania (12.9/100,000) (Angulo et al., 2025). However, epidemiological surveillance data in Poland may underestimate the true burden of the disease. A recent study comparing national surveillance reports with hospitalization data found a discrepancy, suggesting underreporting of TBE cases, particularly during 2020 (Paradowska-Stankiewicz et al., 2023). Several studies suggest that testing of TBE could be improved throughout Poland. Many TBE diagnoses are classified under the generic code A87 of the International Classification of Disease (ICD), which denotes viral encephalitis without a specified viral origin. This results in an underestimation of the disease’s prevalence (Paradowska-Stankiewicz et al., 2023; Zajkowska et al., 2025). Moreover, studies of individuals with lab confirmed cases found that vaccine effectiveness was very high, breakthrough cases are exceptionally rare, and vaccination is currently the only way to prevent disease (Sulik-Wakulińska, Toczyłowski & Grygorczuk, 2025). Although *Ixodes ricinus* is the most common vector of TBEV in Europe (Gritsun, Lashkevich & Gould, 2003), vector competence of *Dermacentor reticulatus* in TBEV transmission has also been confirmed (Ličková et al., 2020). TBEV positive ticks have been found in *D. reticulatus* in Poland (Biernat et al., 2014) and transstadial persistence of TBEV in *D. reticulatus* has been shown (Karbowiak et al., 2016). The range of *D. reticulatus* is expanding and recently was more commonly found on domestic animals, livestock and wild ruminants in central and eastern Poland compared to *I. ricinus* (Mierzejewska et al., 2015b). In certain regions, the prevalence of TBEV may be higher in *D. reticulatus* compared to *I. ricinus*. This was seen in Eastern Poland where TBEV prevalence detection by PCR was 7.6% in *D. reticulatus* (Mierzejewska et al., 2015a). Another study in Eastern Poland found a TBEV minimum infection rate of 1.6% in *I. ricinus* and 10.8% in *D. reticulatus* (Wójcik-Fatla et al., 2011). Understanding the fine-scale geographic distribution and genetic diversity of TBEV aids in the continued monitoring of public health risks and vaccination recommendations. We report the detection and genomic characterization of TBEV in *D. reticulatus* ticks and no detection of TBEV in *I. ricinus* ticks collected in northeastern Poland.

## Materials and Methods

Tick surveillance was performed in military training areas across three regions of Poland. Surveillance was conducted as single site visits conducted in May 2022 and repeated site visits in September 2022. Collection efforts were timed to coincide with the bimodal spring and fall peaks of tick activity. These sites were selected due to their high levels of outdoor activity by military personnel, a population with occupational risk for tick-borne diseases, and access was coordinated through existing military public health surveillance programs. Ticks were collected by flagging for an average of 25 min per site (range: 10–100 min) in diverse habitats, including overgrown fields, forest edges, and two distinct forest classifications: coniferous (pine) forests with sparse undergrowth and deciduous forests with dense undergrowth. Ticks were killed at −20 °C for 24 h and placed in 2 mL tubes of DNA/RNA Shield and transported at −20 °C during the collection trip, but at room temperature during return flight. Once back at the lab, tubes were kept at −80 °C until identification. All collected life stages and sexes of *I. ricinus* and nymphs and adults of *D. reticulatus* were morphologically identified to the species level using a standard taxonomic key for ticks of Europe and North Africa (Estrada-Peña, Mihalca & Petney, 2018). Ticks were thawed for species, life stage and sex identification and kept on ice packs to reduce temperature. Ticks were combined in pools of *N* = 1 to *N* = 4 for adults, *N* = 1 to *N* = 9 for nymphs, and *N* = 3 to *N* = 12 for larva. Males and females were pooled separately, and pooling was performed to be able to sequence more overall ticks. During pooling, ticks were retubed without DNA/RNA shield and stored at −80 °C and shipped on dry ice from WRAIR-EME Georgia to WRAIR VDP in Silver Spring, MD USA. Ticks were homogenized using the MP Biomedicals FastPrep-24 5G with 2 mL lysing matrix A system. Nucleic acid purification, metagenome sequencing, and sequence-based detection of viruses were conducted using a previously described protocol (Li et al., 2025). The tick pool homogenate was inactivated with TRIzol, and RNA was extracted using the PureLink RNA mini kit (Thermo Scientific, Waltham, MA, USA). The Illumina RNA prep with enrichment, along with the Illumina Viral Surveillance Panel, v1, were used to generate a library sequenced on an Illumina MiSeq using a V2 300-cycle kit. Data was processed through the metagenomic pipeline for viral pathogen identification, path-discov (Kilianski et al., 2015) to identify viral pathogens present. Sequences are quality filtered, mapped to the host for additional filtering, and assembled into contigs. Contigs and reads are iteratively aligned to identify known and novel pathogens. Reference-guided genome assembly was performed on these reads against the NCBI RefSeq TBEV genome (NCBI accession: NC_001672.1) using ngs-mapper (https://github.com/VDBWRAIR/ngs_mapper) and manually curated. This new TBEV genome was placed into phylogenetic context by obtaining *N* = 544 full-length (>80% complete) TBEV genomes and two outgroup sequences (Powassan virus and Karshi virus) from BVBRC (Olson et al., 2023) on June 18, 2025. Multiple sequence alignment was performed using MAFFT v7.475 (Katoh & Standley, 2013) (–localpair –maxiterate 1,000 –keeplength –addfragments) with NCBI accession NC_001672.1 (Wallner et al., 1995; Minh et al., 2020) used as a reference. Phylogenetic analysis was performed using IQ-TREE version 1.6.12 (Minh et al., 2020), with 10,000 ultrafast bootstrap replicates and a best fit model of GTR+F+R10. The phylogenetic tree was visualized with FigTree. Recombination analysis was performed using RDP4 v.4.101 (Martin et al., 2015).

## Results

**Figure 1:**
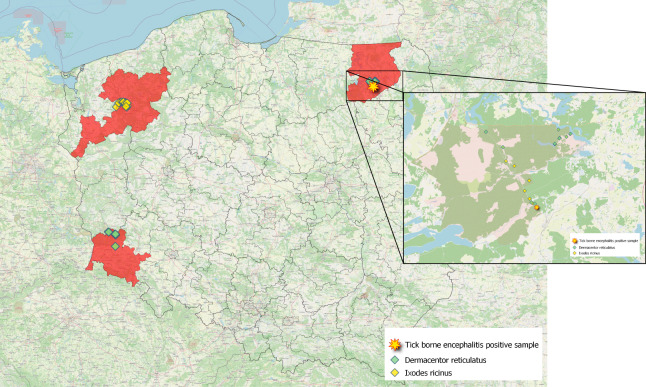
Map of tick collection sites in Poland. *D. reticulatus* collection sites are shown in green; *I. ricinus* collection sites are shown in yellow, and the TBEV-positive pool collection site is labeled with a star. Inset map shows detailed collection locations in northeastern Poland. Map generated with QGIS (QGIS Development Team, 2025) and Eurostat (European Commission, 2025).

Active tick surveillance was conducted in May 2022 in military training areas in Poland (Fig. 1). *D. reticulatus* and *I. ricinus* ticks were collected in forest areas (*N* = 227 ticks), forest edges (*N* = 113 ticks), un-mowed fields (*N* = 74 ticks) and roadsides (*N* = 4 ticks). Most ticks collected were adult (*N* = 197), followed by nymphs (*N* = 168), and larva (*N* = 53) (Table 1).

**Table 1:** Distribution of collected ticks by species, habitat and life stage. A total of 418 ticks were collected, 89 belonging to *D. reticulatus* and 329 to *I. ricinus*. 106 total pools were extracted, consisting of *D. reticulatus*; (*N* = 89 ticks, *N* = 30 pools) and *I. ricinus*; (*N* = 329 ticks, *N* = 76 pools) and were sequenced (Fig. 1 and Table S1). TBEV was detected in one pool of *N* = 3 adult *D. reticulatus* ticks collected in a forest. *Rare *D. reticulatus* nymphs were collected in a forest site that was in a heavily vegetated path.

Life stage	Forest	Forest–Edge	Field–Un-mowed	Roadside	Total
***Ixodes ricinus***					
**Adult**	54	38	19	0	**111**
**Nymph**	99	34	28	4	**165**
**Larva**	39	14	0	0	**53**
**Total**	**192**	**86**	**47**	**4**	**329**
***Dermacentor reticulatus***					
**Adult**	32	27	27	0	**86**
Nymph*	3	0	0	0	**3**
**Larva**	0	0	0	0	**0**
**Total**	**35**	**27**	**27**	**0**	**89**
**All species**					
**Adult**	86	65	46	0	**197**
**Nymph**	102	34	28	4	**168**
**Larva**	39	14	0	0	**53**
**Total**	**227**	**113**	**74**	**4**	**418**

**Figure 2:**
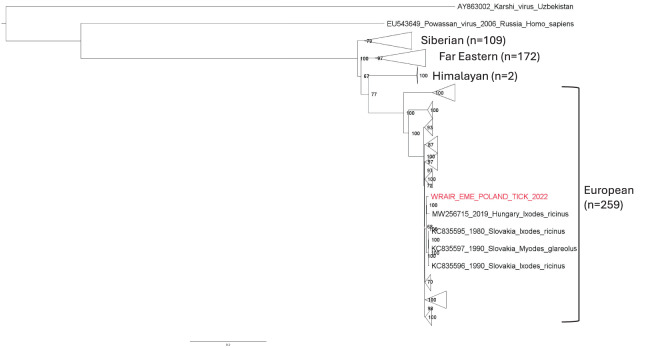
Phylogenic placement of new TBEV full genome from *D. reticulatus* ticks. Phylogenetic tree of TBEV genome WRAIR_EME_POLAND_TICK_2022 with TBEV subtype and genotypes listed. Nodes have been collapsed into triangles for visualization purposes. Node values represent percentage of ultrafast bootstrap replicate support. The entire expanded tree of *N* = 544 samples is provided in Fig. S1.

Initial shotgun sequencing of the TBEV positive *D. reticulatus* pool resulted in sequencing reads covering 3,034 bp of the 11,173 bp reference genome (27% of NC_001672.1). Subsequent hybridization-enrichment library preparation and sequencing resulted in 99% of this TBEV genome being assembled with a minimum depth of coverage of 20 reads, and an average sequencing depth of 702×. No evidence of recombination was detected in this genome. Phylogenetic analysis placed the TBEV genome (designated WRAIR_EME_POLAND_TICK_2022) within the European subtype clade with 100% bootstrap support (Fig. 2 and Fig. S1). The strain was most closely related to a Hungarian isolate, MW256715 (Egyed et al., 2021).

## Discussion

This study documents the detection of TBEV in *D. reticulatus* ticks in northeastern Poland, providing a more detailed geographic presence of TBEV in the region. The minimum infection rates of TBEV of 1.1% in *D. reticulatus* and 0.0% in *I. ricinus* in this study are comparable to other studies in Poland, although slightly lower than the minimum infection rate reported for *I. ricinus* (1.6%) and *D. reticulatus* (10.8%) (Wójcik-Fatla et al., 2011) and prevalence of TBEV *D. reticulatus* in (7.6%) (Mierzejewska et al., 2015a). The 10.8% TBEV prevalence in *D. reticulatus* in the Lublin region of Poland (Wójcik-Fatla et al., 2011) is approximately 300 km south of the TBEV positive collection site in this study. The 7.6% TBEV prevalence in *D. reticulatus* in endemic regions and zones of expansion in Poland (Mierzejewska et al., 2015a) is difficult to compare to this study due to ambiguity in reporting, but the majority (six of seven positive ticks) were collected in the Masovian Voivodeship which does not overlap with our positive TBEV pool collected further northeast. There may be several reasons accounting for these differences including exact geographical location variation, sensitivity of PCR *vs*. shotgun metagenomic sequencing approaches, and surveillance design. Additionally, it is possible, though unlikely that the TBEV positive pool presented here contained more than one positive tick, which would increase the minimum infection rate for *D. reticulatus* in this study. The assembled TBEV genome represents the first full-length TBEV genome sequenced from *D. reticulatus* ticks. The identification of the European subtype is consistent with previous findings in other parts of Poland (Biernat et al., 2014; Paradowska-Stankiewicz et al., 2023). The complete genome sequence provides valuable data for understanding the genetic diversity and evolution of TBEV. The close genetic relationship between the Polish TBEV genome assembled here to a Hungarian sample from nearly 1,000 km away may offer insight into the virus’s transmission dynamics. These phylogenetic results recapitulate previous studies that show close genetic relationships among TBEV viruses collected from dispersed geographical distances (Deviatkin et al., 2020). Such long-distance genetic linkage cannot be explained by tick dispersal alone and it implicates the central role of mobile vertebrate hosts in the TBEV ecology. Major avian migratory flyways go through Poland and migratory birds infested with *I. ricinus, D. reticulatus* and other species have been identified in Poland and suggest a mechanism for viral dispersal (Ciebiera et al., 2019; Michelitsch et al., 2019; Buczek et al., 2020; Zając et al., 2022). This includes landscape corridors, biogenic factors (migratory birds and large mammals) as well as anthropogenic transport (Weidmann et al., 2013). The long distance implicates the role of mobile animal hosts in this movement and warrants further investigation. Historically, TBEV has been primarily associated with *I. ricinus* ticks (Beauté et al., 2018). This and other recent studies finding TBEV in *D. reticulatus* ticks (Ličková et al., 2020; Król et al., 2024) may represent a potential shift in vector ecology and/or disease transmission dynamics that could be further investigated and monitored. Also of note is the finding of three nymph stage *D. reticulatus* ticks collected in a forest site that was in a heavily vegetated path which is particularly rare especially using standard flagging methods of collection. A recent study also found the rare presence of active host-seeking *D. reticulatus* nymphs collected in February 2024 in Nowy Staw, Poland (Buczek et al., 2025). This collection location was approximately 210 km from the *D. reticulatus* nymph collection site (and adult TBEV positive tick in this study) in a rural recreation area with mixed forest, meadows, clearings, marshes, peat bogs, and artificial ponds. Continued surveillance will help to better determine the prevalence of TBEV in additional tick species, collected across additional time periods, and diverse habitat types to best assess the risk of human infection in this region and best inform ongoing effective TBEV vaccination strategies.

## Conclusions

TBEV is clinically important virus that can cause a wide range of indicators from asymptomatic cases to cases with neurological symptoms such as meningitis, encephalitis, and meningoencephalitis. Case fatality rates of TBEV infection range from 2–40% depending on subtype. Many patients can experience long term neurological sequelae. The primary mode of infection in humans is through tick bites. This study surveilled *I. ricinus* and *D. reticulatus* ticks collected from military training sites in several locations in Poland during the 2022 season. Shotgun metagenomics was performed on 106 pools of 418 ticks and resulted in one TBEV positive pool of *D. reticulatus* ticks. This positive pool was further characterized to produce the first full length genome sequence of TBEV from a *D. reticulatus* tick. The minimum infection rates of these two tick species in this collection are comparable, but slightly lower than other similar reports in Poland. Phylogenetic analysis of the TBEV genome revealed a close genetic relationship to a TBEV isolate genome collected in Hungary nearly 1,000 km away. This study highlights the continued importance of tick pathogen surveillance in endemic regions such as Poland.

## Supplemental Information

10.7717/peerj.21588/supp-1 Supplemental Information 1 Expanded phylogenetic tree. Entire expanded phylogenetic tree (*N* = 544 samples) and *D. reticulatus* TBEV genome WRAIR_EME_POLAND_TICK_2022. Node values represent percentage of ultrafast bootstrap replicate support.

10.7717/peerj.21588/supp-2 Supplemental Information 2 Tick sample collection details. Detailed tick collection information including location, date, habitat, species, life stage, vector count of pools and NCBI accession IDs for *I. ricinus* and *D. reticulatus* collected.

## Additional Information and Declarations

### Data Availability

The following information was supplied regarding data availability:

The data is available in the Supplemental Files.

The raw sequence data are available at NCBI: PZ157927, PRJNA1438251.
