# Supplementary material for: Tick-borne encephalitis virus in Dermacentor reticulatus ticks, Poland
Source: PeerJ. 2026 Aug 10;14:e21588. doi: 10.7717/peerj.21588 (PMC13471749; doi:10.7717/peerj.21588)

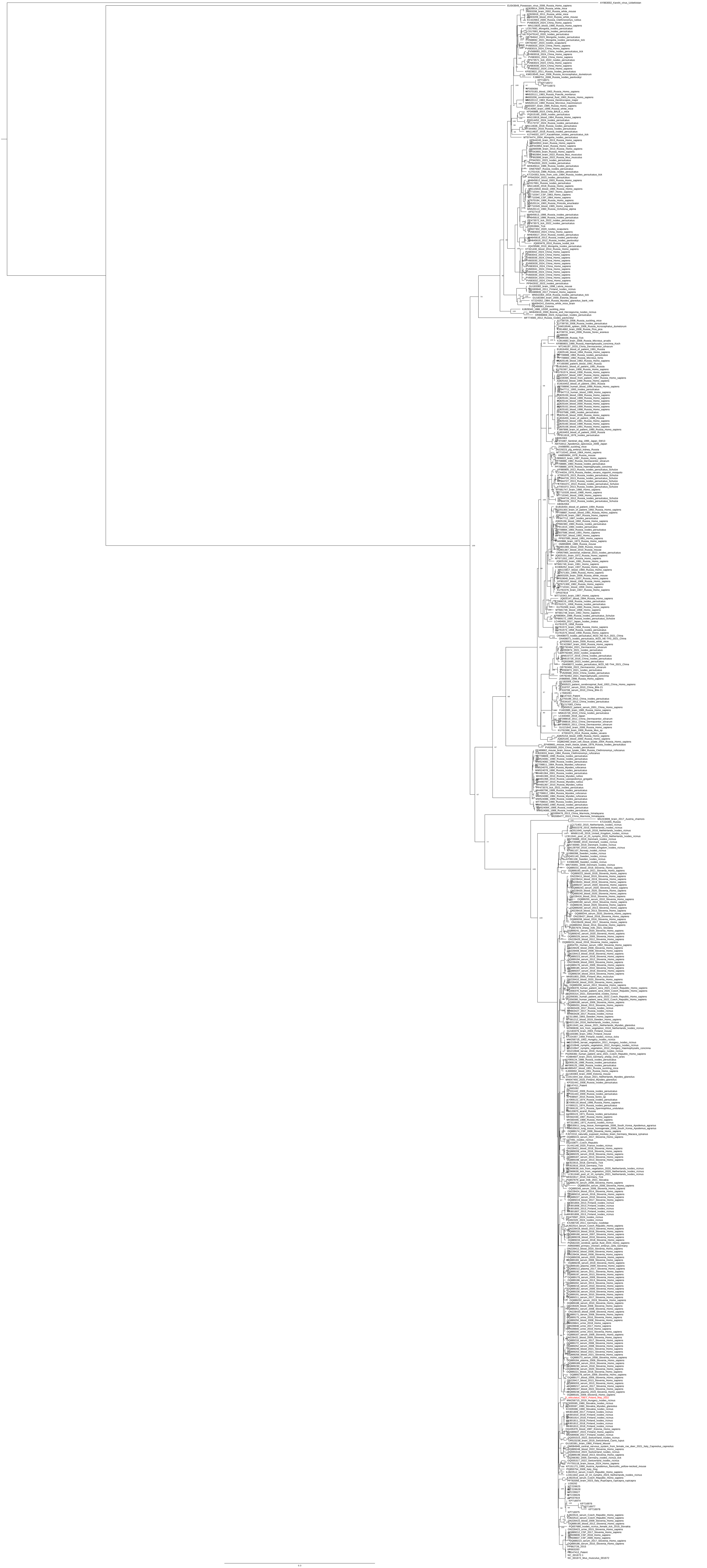

Supplement: Supplementary file 1 [file peerj-14-21588-s001.pdf]
